# Microparticle-induced release of B-lymphocyte regulators by rheumatoid synoviocytes

**DOI:** 10.1186/ar2648

**Published:** 2009-03-16

**Authors:** Laurent Messer, Ghada Alsaleh, Jean-Marie Freyssinet, Fatiha Zobairi, Isabelle Leray, Jacques-Eric Gottenberg, Jean Sibilia, Florence Toti-Orfanoudakis, Dominique Wachsmann

**Affiliations:** 1 Laboratoire Physiopathologie des Arthrites, Université de Strasbourg, UFR Sciences Pharmaceutiques, 74 route du Rhin, Illkirch 67401, France; 2 Département de Rhumatologie, Hôpitaux Universitaires de Strasbourg, Avenue Molière, Strasbourg Hautepierre 67200, France; 3 Laboratoire de Biologie Cellulaire et Vasculaire, Faculté de Médecine, 4 rue Kirschleger, Strasbourg 67085, France; 4 Inserm U770 Hôpital Bicêtre (AP-HP), 78 rue du Général Leclerc, Le Kremlin-Bicêtre 94275, France

## Abstract

**Introduction:**

In the present study, we investigated the ability of microparticles isolated from synovial fluids from patients with rheumatoid arthritis or osteoarthritis to induce the synthesis and release of key cytokines of B-lymphocyte modulation such as B cell-activating factor, thymic stroma lymphopoietin, and secretory leukocyte protease inhibitor by rheumatoid fibroblast-like synoviocytes.

**Methods:**

Microparticles were analyzed in synovial fluids from patients with rheumatoid arthritis, osteoarthritis, microcristalline arthritis, and reactive arthritis. In addition, microparticle release after activation from various cell lines (CEM lymphocyte and THP-1 cells) was assessed. Microparticles were isolated by differential centrifugation, and quantitative determinations were carried out by prothrombinase assay after capture on immobilized annexin V. B cell-activating factor, thymic stroma lymphopoietin, and secretory leukocyte protease inhibitor release was evaluated by enzyme-linked immunosorbent assay.

**Results:**

Microparticles isolated from synovial fluids obtained from rheumatoid arthritis and osteoarthritis patients or microparticles derived from activated THP-1 cells were able to induce B cell-activating factor, thymic stroma lymphopoietin, and secretory leukocyte protease inhibitor release by rheumatoid arthritis fibroblast-like synoviocytes. Conversely, CEM-lymphocytes-derived microparticles generated by treatment with a combination of PHA, PMA and Adt-D did not promote the release of B cell-activating factor but favored the secretion of thymic stroma lymphopoietin and secretory leukocyte protease inhibitor by rheumatoid arthritis fibrobast-like synoviocytes. However, microparticles isolated from actinomycin D-treated CEM lymphocytes were not able to induce B cell-activating factor, thymic stroma lymphopoietin, or secretory leukocyte protease inhibitor release, indicating that microparticles derived from apoptotic T cells do not function as effectors in B-cell activation.

**Conclusions:**

These results demonstrate that microparticles are signalling structures that may act as specific conveyors in the triggered induction and amplification of autoimmunity. This study also indicates that microparticles have differential effects in the crosstalk between B lymphocytes and target cells of autoimmunity regarding the parental cells from which they derive.

## Introduction

Rheumatoid arthritis (RA) is characterized by a disorganized reaction of the inflammatory and synovial resident cells, fibroblast-like synoviocytes (FLSs), which have a key function in the development of inflammation as well as in tissue destruction [1,2]. The activation of the latter may be linked either to the cytokine environment or to interactions between pathogen-associated molecular patterns and pattern-recognition receptors [3,4].

FLSs can also be activated by cell-cell interactions and microparticles (MPs). MPs are submicron structures released from the cell membrane during apoptosis or activation, and they probably play an important role in this intercellular triggering process [5]. MPs expose phosphatidylserine and display surface plasma membrane markers from their parental cells [6]. They are involved in the modulation of key functions, including inflammation, hemostasis, and angiogenesis [7-10]. Elevated MPs circulate in the blood of patients with various inflammatory disorders [11-14]. Berckmans and colleagues [15] demonstrated that, in RA, synovial MPs isolated from arthritic patients induced cytokine release by FLSs. Similarly, Distler and colleagues [16] demonstrated that MPs derived from T cells and monocytes induced the synthesis of various cytokines such as interleukin (IL)-6, IL-8, monocyte chemoattractant protein (MCP)-1 and MCP-2, and metalloproteases such as matrix metalloproteinase (MMP)-1, MMP-3, MMP-9, and MMP-14 by activated FLSs. Thus, MPs appear as multifunctional bioeffectors that could be implicated in the exacerbation of the inflammatory response and cartilage and bone erosion by resident cells in RA.

Previous findings have shown that FLSs participate in the development of the specific immune response by secreting cytokines (SDF-1 and CXCL13) that attracted B cells and allowed the formation of pseudofollicles in the synovial membrane [17-19]. Ohata and colleagues [20] demonstrated that FLSs isolated from RA patients express B cell-activating factor (BAFF) transcripts in response to tumor necrosis factor-alpha and interferon-gamma (IFN-γ). BAFF is known to play a central role in the maturation and survival of B cells as well as in antibody synthesis. However, we recently demonstrated that BAFF secretion by RA FLSs is tightly regulated by a complex network involving innate immunity and cytokines, with positive and negative controls depending on the receptors and pathways triggered. Thus, BAFF synthesis and release by RA FLSs are negatively regulated by Toll-like receptor (TLR) ligands whereas integrin signalling pathways stimulate BAFF secretion by resident cells [21].

It was recently demonstrated that, in the presence of TLR-binding products, epithelial cells from tonsillar crypts released BAFF and IL-10, which stimulated B cells to secrete polyreactive antibodies to multiple microbial determinants. This effect was enhanced by epithelial cell release of thymic stroma lymphopoietin (TSLP), which induced BAFF production by dendritic cells but could also restrain it by secretory leukocyte protease inhibitor (SLPI) release from activated epithelial cells [22]. In the present study, we investigated the capacity of MPs isolated from synovial fluids of RA and osteoarthritis (OA) patients or derived from various activated cell lines to induce the release of BAFF, TSLP, and SLPI by FLSs isolated from RA patients.

Our data indicated that MPs isolated from synovial fluids of OA and RA patients were able to induce BAFF, TSLP, and SLPI release by activated FLSs. Since it had been previously demonstrated that most of the MPs present in the synovial fluid of inflamed joints were leukocyte-derived MPs [23], we investigated the ability of MPs isolated from activated CEM lymphocyte and THP-1 cells to induce BAFF, TSLP, and SLPI synthesis by activated RA FLSs.

MPs derived from activated THP-1 cells induced such release as well. In contrast, those derived from activated CEM lymphocytes did not promote the release of BAFF but favored the secretion of TSLP and SLPI by RA FLSs. However, MPs isolated from actinomycin D (ActD)-treated CEM lymphocytes were not able to induce BAFF, TSLP, and SLPI release, indicating that MPs derived from apoptotic T cells do not function as effectors in B-cell activation. Together, these results indicate that MPs represent signalling structures that may act as inducers and amplifying devices of inflammatory and specific immune responses.

## Materials and methods

### Reagents

Cell culture media (RPMI 1640 and M199), fetal calf serum (FCS), penicillin, streptomycin, and amphotericin B were obtained from Invitrogen Corporation (Cergy-Pontoise, France). Human recombinant IFN-γ was purchased from BD Pharmingen (Le Pont-de-Claix, France). Lipopolysaccharide (LPS) from *Salmonella abortus equi *and type XI collagenase, Hanks' balanced saline solution (HBSS), ActD, phorbolmyristate acetate (PMA), and phytohemagglutinin (PHA) were obtained from Sigma-Aldrich (Saint-Quentin-Fallavier, France). The enzyme immunoassay kits for human BAFF, TSLP, SLPI, IL-6, and IL-8 detection were from R&D Systems (Lille, France).

### Cell culture

Human FLSs were isolated from RA synovial tissues from different patients at the time of knee joint arthroscopic synovectomy as described previously [24]. The diagnosis conformed to the revised criteria of the American College of Rheumatology [25]. Informed consent was provided in accordance with the Declaration of Helsinki and obtained from all patients. Approval by the ethics committee of the Hopitaux Universitaires de Strasbourg was obtained. FLS cultures were performed as previously described [26]. Experiments were performed between the third and ninth passages. During that time, cultures were constituted of a homogeneous population of fibroblastic cells that were negative for CD16 as determined by fluorescence-activated cell sorting analysis. Cell number and cell viability were checked by the MTT (3-(4,5 dimethylthiazol-2-yl)-2,5-diphenyltetrazolium bromide) test as described elsewhere [27]. The THP-1 monocyte cell line and CEM lymphocytes were obtained from the American Type Culture Collection (Manassas, VA, USA).

### *In vitro *generation of microparticles and isolation from cell and plasma samples

The release of MPs from CEM lymphocyte cells was induced by stimulation either with ActD alone at a concentration of 0.5 μg/mL for 18 hours or with a combination of PHA (5 μg/mL) for 72 hours followed by PMA (20 ng/mL) and ActD (0.5 μg/mL) for an additional 18-hour incubation period. THP-1 cells were treated with LPS (15 μg/mL) for 18 hours. Cell culture supernatants were centrifuged at 400 *g *for 5 minutes and then at 750 *g *for 15 minutes. Supernatants were harvested and centrifuged at 17,000 *g *for 30 minutes at 4°C. Pellets were washed in HBSS, centrifuged for 30 minutes at 17,000 *g *at 4°C, and finally resuspended in 500 μL of HBSS. Synovial fluids were collected from RA, OA, microcristalline arthritis (MC), and reactive arthritis (AR) patients on sodium citrate (0.129 M) and centrifuged at room temperature for 15 minutes at 1,500 *g *and then for 2 minutes at 13,000 *g*. Supernatants were stored at -80°C until use. In FLS-mediated activation experiments, MPs were isolated from patients by differential centrifugation as described for CEM lymphocyte and THP-1 cells. The last supernatant was used as a negative control to ensure that no remaining proteins could be responsible for the observed MP-mediated effects. Quantitative determinations of MPs were carried out using a prothrombinase assay after capture on immobilized annexin V as previously described. MP values are expressed as phosphatidylserine equivalents (PhtdSer Eq) by reference to a calibration curve constructed with synthetic phospholipid vesicles [28].

### Stimulation of cells for cytokine assays

RA FLSs (2 × 10^5 ^cells) were stimulated with 1 mL of complete medium (RPMI 1640 and M199/5% dialyzed FCS) containing MPs. BAFF and SLPI secretion was assessed after 72-hour MP treatment, whereas SLPI was measured after 48-hour MP treatment by a heterologous two-site sandwich enzyme-linked immunosorbent assay (ELISA) in accordance with the instructions of the manufacturer (R&D Systems, Lille, France). FLSs (5 × 10^3^ cells) were grown to confluence in 96-well plates (7 to 10 days) and then stimulated with 200 μL of serum-free RPMI 1640/M199 containing MPs. After a 20-hour incubation period, a heterologous two-site sandwich ELISA was used to estimate IL-6 and IL-8 release in culture supernatants. Negative controls consisted of cell culture medium and last supernatants obtained after MP isolation.

### Statistical analysis

Results are expressed as mean ± standard deviation. Statistical analysis was carried out using the Student test and by Wilcoxon non-parametric test to compare mean values between patient values or secreted molecules and released MPs in each experiment. All analyses were performed using SPSS 13.0 software (SPSS Inc., Chicago, IL, USA).

## Results

### Isolation of microparticles

**Figure 1:**
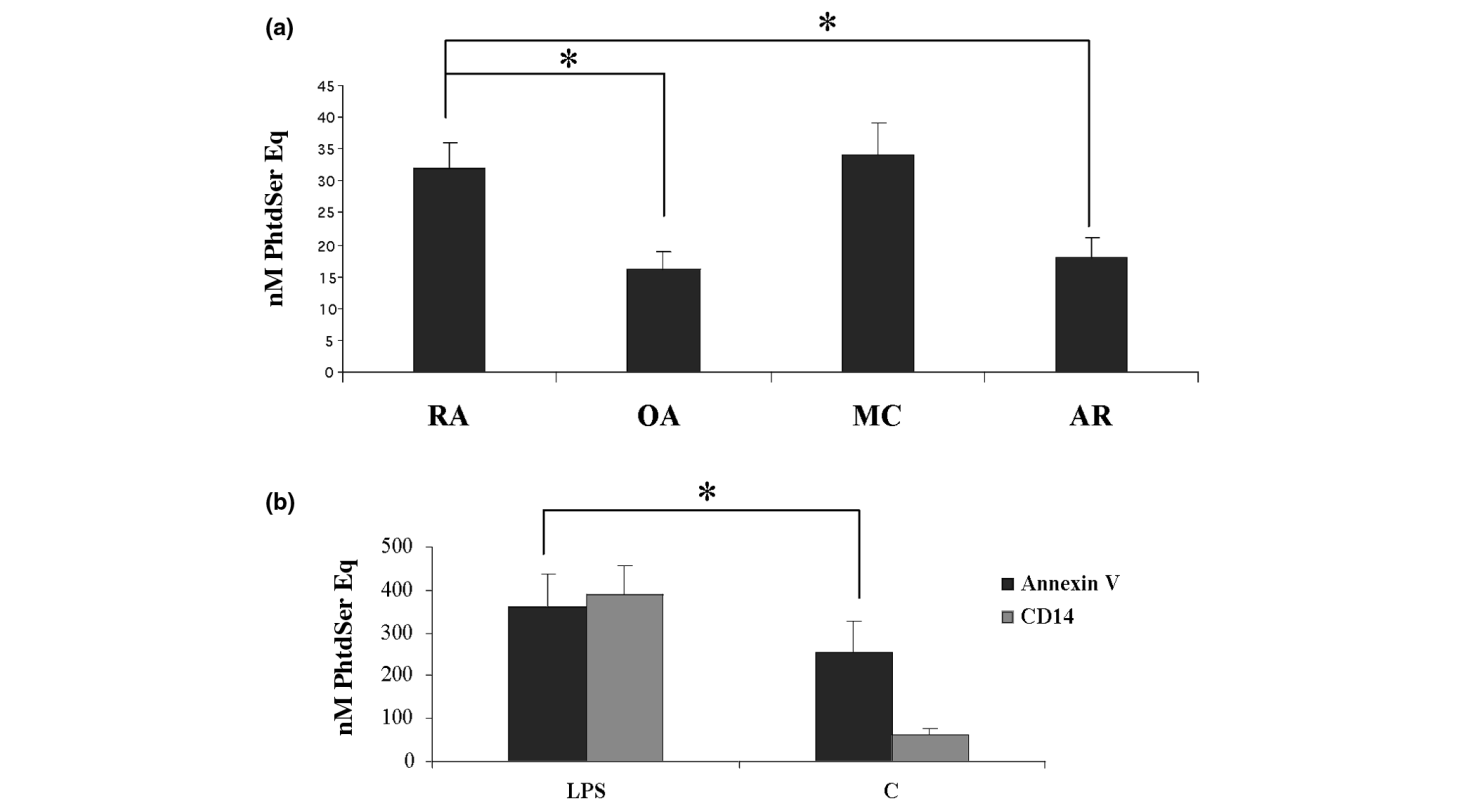
Microparticle (MP) assessment in synovial fluids from arthritic patients and cell supernatants. **(a) **Concentrations of MPs in synovial fluids from patients with rheumatoid arthritis (RA) (n = 7), osteoarthritis (OA) (n = 5), microcristalline arthritis (MC) (n = 3), and reactive arthritis (AR) (n = 5) were determined by a solid-phase capture assay on immobilized annexin V by use of a prothrombinase assay (nM PhtdSer Eq). **(b) **Concentrations of MPs isolated from THP-1 cells stimulated with lipopolysaccharide (LPS) (15 μg/mL) for 18 hours were determined by a solid-phase capture assay on immobilized annexin V or on immobilized CD14 antibody by use of a prothrombinase assay (nM PhtdSer Eq). The control (C) corresponded to untreated cells. Data are expressed as the mean of triplicate samples ± standard deviation and are representative of three independent experiments. *P < 0.05. PhtdSer Eq, phosphatidylserine equivalents.

MPs were isolated from synovial fluids obtained from patients with RA (n = 7), OA (n = 5), MC (n = 3), and AR (n = 5) by differential centrifugation. Characteristics of the patients are presented in Table 1. In this system, exosomes were eliminated after the first centrifugation at 17,000 *g*. Quantitative determinations of MPs were carried out using a prothrombinase assay after capture on immobilized annexin V [28]. As shown in Figure 1a, synovial fluids from all patients tested contained MPs but their number was significantly higher in synovial fluids isolated from RA and MC patients (32 ± 4 and 34 ± 5 nM PhtdSer Eq) compared with levels measured in synovial fluids obtained from OA and AR patients (16 ± 3 and 18 ± 3 nM PhtdSer Eq, respectively). We also performed activation experiments with MPs isolated from CEM lymphocyte and THP-1 cells. CEM lymphocyte cells were treated with ActD (0.5 μg/mL) or with a combination of PHA (5 μg/mL), ActD (0.5 μg/mL), and PMA (20 ng/mL) as described in Materials and methods. THP-1 cells were stimulated with LPS from *Salmonella abortus equi *(15 μg/mL) and MPs were then isolated and quantified as described in Materials and methods. MP capture assay on annexin V showed that LPS treatment increased the number of MPs released from activated THP-1 cells (362 ± 76 nM PhtdSer Eq) as compared with untreated THP-1 cells (254 ± 69 nM PhtdSer Eq) (*P *< 0.05). MP capture on immobilized CD14 antibody indicated that stimulated THP-1 cells significantly released a higher proportion of MPs bearing CD14 (390 ± 75 nM PhtdSer Eq) than unstimulated cells (63 ± 12 nM PhtdSer Eq) (Figure 1b).

**Table 1:** Characteristics of patients

Diagnosis	Patient	Gender	Age, years	Disease duration, years	Disease activity	Medications
RA	1	Male	75	10	DAS28 = 3.9 CRP = 19 mg/L	Methotrexate 15 mg Prednisone 5
	2	Female	86	5	DAS28 = 4.7 CRP = 12 mg/L	Methotrexate 10 mg Prednisone 7 mg
	3	Female	79	20	DAS28 = 6 CRP = 78 mg/L	Methotrexate 15 mg Prednisone 10 mg
	4	Female	46	15	DAS28 = 4.1 CRP = 17 mg/L	Methotrexate 10 mg Prednisone 2 mg
	5	Female	55	6	DAS28 = 3.7 CRP = 30 mg/L	Methotrexate 17.5 mg Prednisone 5 mg
	6	Male	75	2	DAS28 = 4.2 CRP = 67 mg/L	Methotrexate 10 mg Prednisone 7 mg
	7	Male	46	15	DAS28 = 3.6 CRP = 150 mg/L	Methotrexate 10 mg Prednisone 5 mg Infliximab (3 mg/kg)

OA	1	Male	54	8	CRP = 4 mg/L	Paracetamol, NSAID
	2	Female	56	3	CRP = 6 mg/L	Paracetamol
	3	Female	46	5	CRP = 4 mg/L	Paracetamol, NSAID
	4	Female	65	7	CRP = 6 mg/L	Paracetamol
	5	Male	55	5	CRP = 4 mg/L	Paracetamol, Tramadol

MC	1	Female	55	3	CRP = 80 mg/L	Paracetamol, NSAID
	2	Male	50	1	CRP = 34 mg/L	Colchicine
	3	Male	74	1	CRP = 90 mg/L	NSAID

AR	1	Male	75	1	CRP = 54 mg/L	NSAID
	2	Male	30	2	CRP = 82 mg/L	NSAID
	3	Male	45	1	CRP = 20 mg/L	NSAID
	4	Female	20	1	CRP = 34 mg/L	Paracetamol
	5	Male	52	1	CRP = 25 mg/L	Paracetamol

Patients were diagnosed with rheumatoid arthritis based on American College of Rheumatology 1987 diagnostic criteria at least 3 months before study entry and have active disease. The diagnosis of microcristalline arthritis was based on clinical and laboratory features. All patients fulfilled the American College of Rheumatology criteria for acute gouty or pseudogout. All patients gave informed consent. AR, reactive arthritis; CRP, C-reactive protein; DAS28, disease activity score using 28 joint counts; MC, microcristalline arthritis; NSAID, nonsteroidal anti-inflammatory drugs; OA, osteoarthritis; RA, rheumatoid arthritis.

### Microparticles promoted the synthesis of B cell-activating factor by activated rheumatoid arthritis fibroblast-like synoviocytes

**Figure 2:**
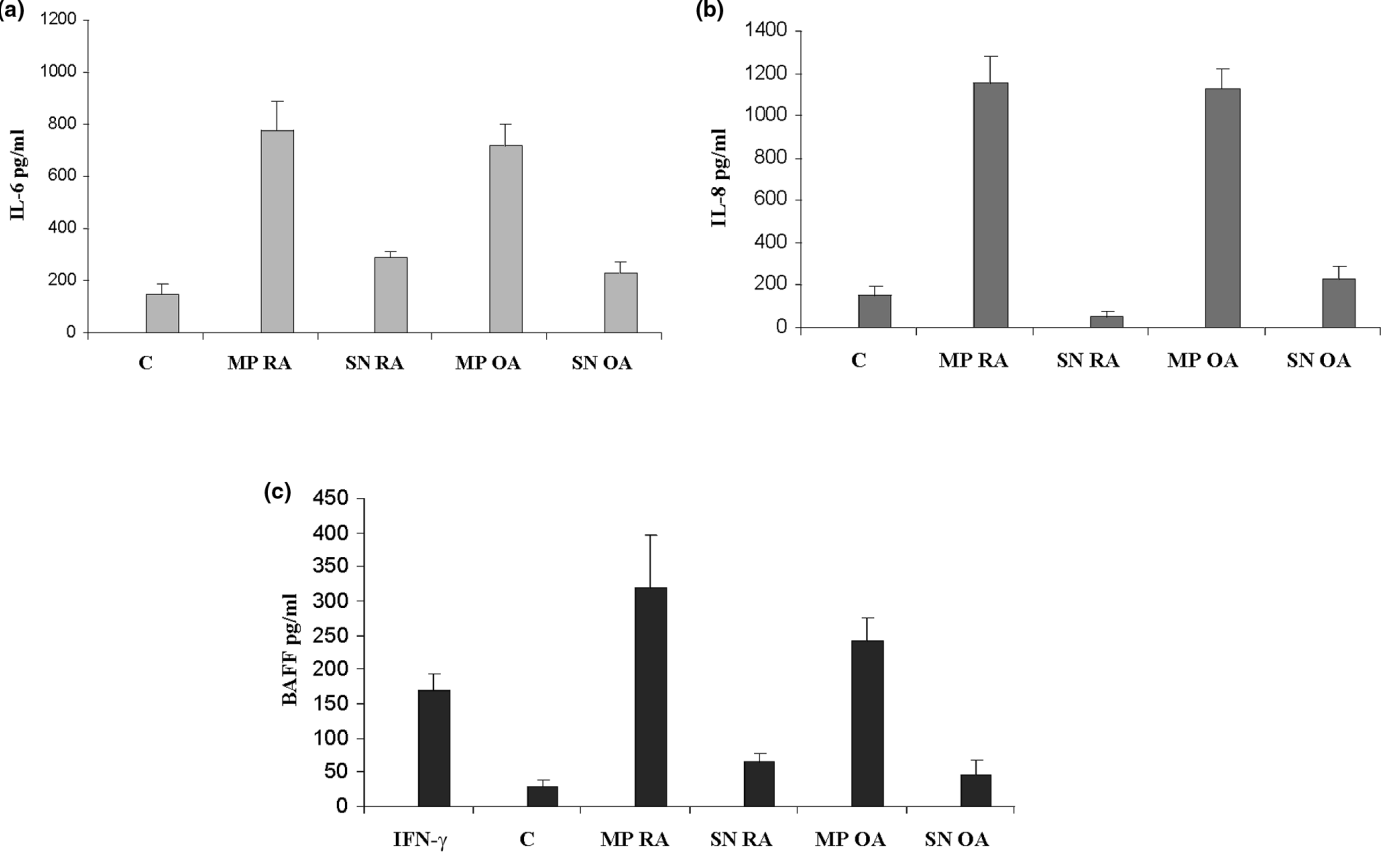
Induction of interleukin (IL)-6, IL-8, and B cell-activating factor (BAFF) synthesis by microparticles (MPs) isolated from synovial fluids. Rheumatoid arthritis (RA) fibroblast-like synoviocytes were stimulated with 40 nM phosphatidylserine equivalents of MPs isolated from synovial fluids of osteoarthritis (OA) and RA patients for 24 and 72 hours. IL-6 **(a)**, IL-8 **(b)**, and BAFF **(c) **release was determined by enzyme-linked immunosorbent assay. Data are expressed as the mean of triplicate samples ± standard deviation and are representative of three independent experiments. C, control medium; IFN-γ, interferon-gamma; SN, control supernatants.

To determine whether MPs are biologically active, we evaluated their ability to induce BAFF release by activated RA FLSs. In parallel, we also determined IL-6 and IL-8 production by MP-treated RA FLSs. FLSs were incubated for 20 and 72 hours with MPs isolated from RA synovial fluids, and IL-6, IL-8, and BAFF release was determined by ELISA. As shown in Figure 2a,b, incubation of FLSs with MPs at a concentration of 40 nM PhtdSer Eq increased IL-6 and IL-8 release by RA FLSs. IL-6 and IL-8 release reached 770 ± 110 and 1,150 ± 130 pg/mL, respectively, after 20-hour incubation as compared with control medium (145 ± 40 and 150 ± 46 pg/mL) and control supernatants (300 ± 30 and 50 ± 23 pg/mL) (Figure 2a,b). Equal concentrations of OA MPs had a comparable effect on IL-6 and IL-8 release.

We next determined whether MPs isolated from OA and RA synovial fluids might also play a role in BAFF release by FLSs. RA FLSs were incubated for 72 hours with MPs used at concentrations mentioned above, and BAFF release was evaluated in culture supernatants by ELISA. As shown in Figure 2c, BAFF secretion was increased in response to either OA or RA MPs after 72 hours and reached 320 ± 76 pg/mL in response to RA MPs and 250 ± 34 pg/mL in response to OA MPs as compared with control medium (30 ± 9 pg/mL) and control supernatants (70 ± 12 and 50 ± 21 pg/mL, respectively). IFN-γ (180 ± 23 pg/mL) was used as a positive control. Taken together, these results indicate that MPs isolated from either OA and RA synovial fluids were able to activate IL-6, IL-8, and BAFF release by activated FLSs.

It was previously demonstrated that most MPs present in the synovial fluid of inflamed joints were leukocyte-derived MPs [23]. In the present study, we investigated the ability of MPs isolated from activated CEM lymphocyte and THP-1 cells to induce BAFF synthesis by activated RA FLSs.

**Figure 3:**
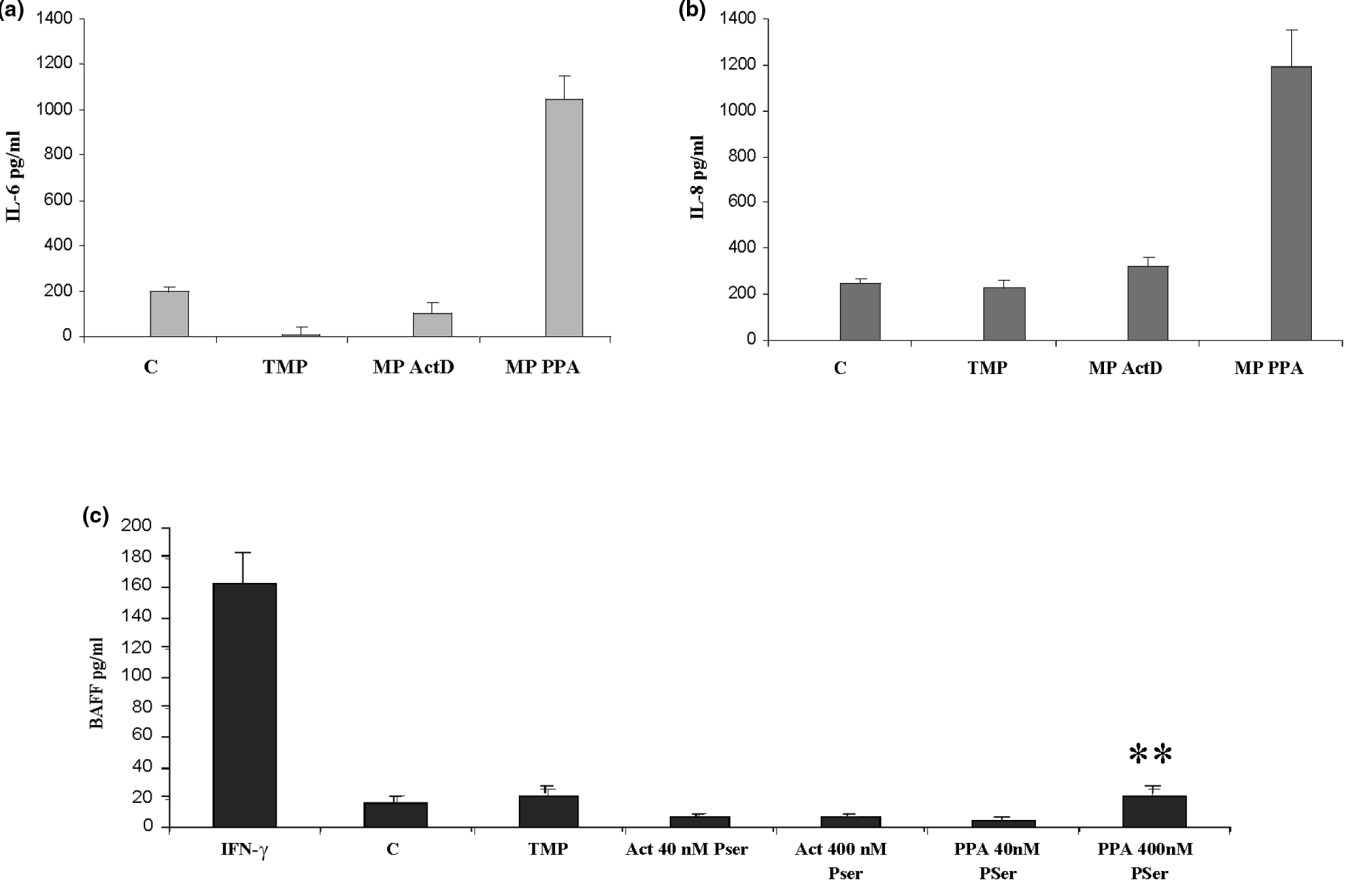
Induction of interleukin (IL)-6, IL-8, and B cell-activating factor (BAFF) synthesis by microparticles (MPs) isolated from CEM lymphocytes. Rheumatoid arthritis fibroblast-like synoviocytes were stimulated with 40 and 400 nM phosphatidylserine equivalents of MPs isolated from CEM lymphocytes treated either with actinomycin D (ActD) alone (0.5 μg/mL) for 18 hours or with a combination of PHA, PMA and Adt-D (PPA) (phytohemagglutinin) (5 μg/mL) for 72 hours followed by ActD (0.5 μg/mL) and phorbolmyristate acetate (20 ng/mL) for an additional 18-hour incubation period. Culture supernatants were harvested 24 hours after stimulation for IL-6 **(a) **and IL-8 **(b) **determination and 72 hours after stimulation for BAFF evaluation **(c) **by enzyme-linked immunosorbent assay. Lipopolysaccharide (IL-6 and IL-8) and interferon-gamma (IFN-γ) (BAFF) stimulation was used as a positive control. Data are expressed as the mean of triplicate samples ± standard deviation and are representative of three independent experiments. **P < 0.01. Act, actinomycin; C, control medium; Pser, phosphatidylserine; TMP, control Hanks' balanced saline solution.

IL-6 and IL-8 release was enhanced by MPs from ActD/PMA/PHA-treated CEM lymphocytes (1,050 ± 100 and 1,200 ± 210 pg/mL for IL-6 and IL-8, respectively) when MPs were used at the concentration of 40 nM PhtdSer Eq (Figure 3a,b). Such MPs did not increase BAFF production at the same concentration. No induction of BAFF release was observed with a 10-fold increase in MP concentration (*P *< 0.01). IFN-γ (170 ± 25 pg/mL) was used as a positive control of BAFF secretion (Figure 3c). Similar concentrations of MPs derived from ActD-treated CEM lymphocytes exhibited no significant effect on IL-6, IL-8, or BAFF release by activated RA FLSs (Figure 3c).

**Figure 4:**
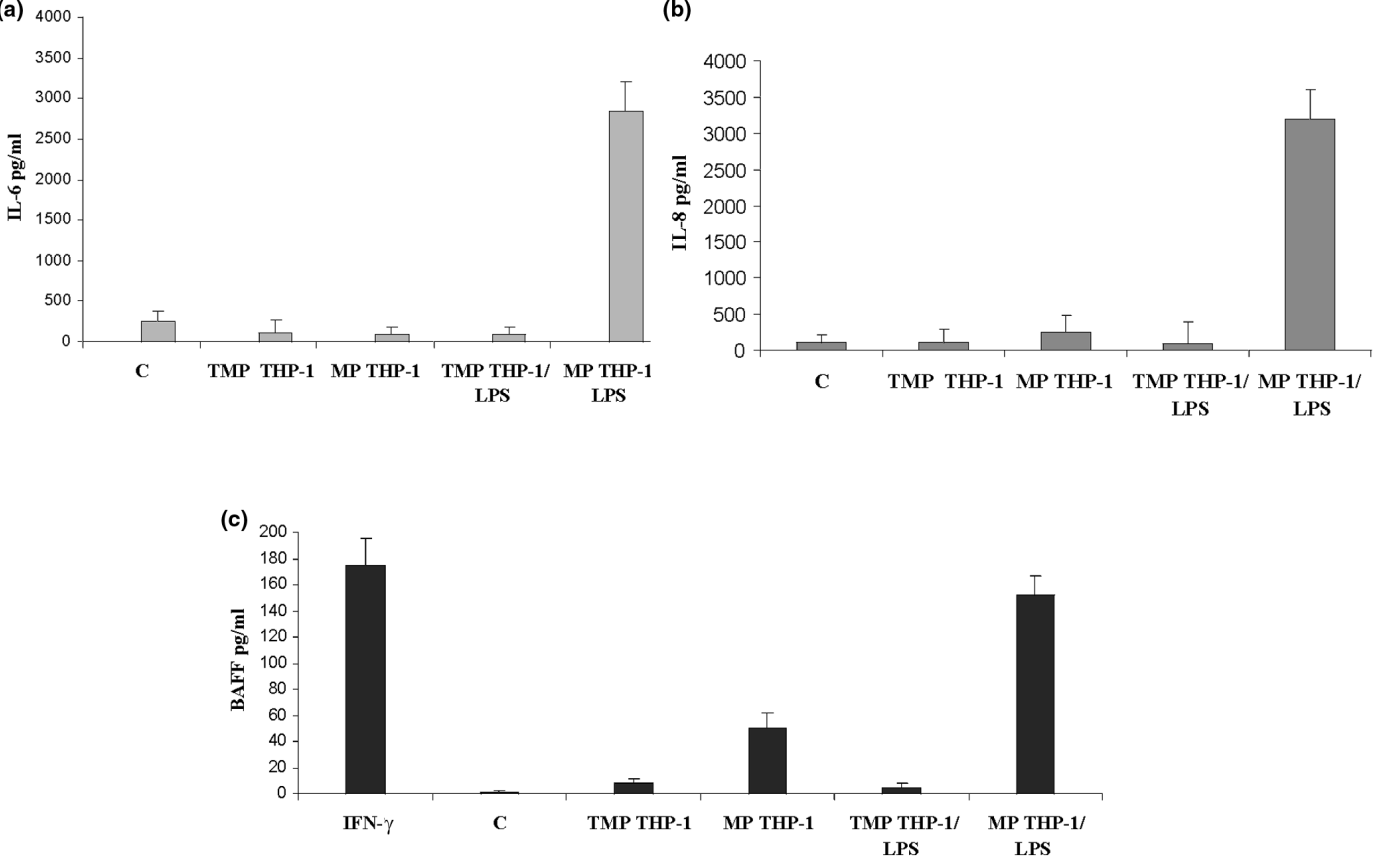
Induction of interleukin (IL)-6, IL-8, and B cell-activating factor (BAFF) synthesis by microparticles (MPs) isolated from lipopolysaccharide (LPS)-activated THP-1 cells. THP-1 cells were treated with LPS (15 μg/mL) for 18 hours, and MPs were isolated as described in Materials and methods. Rheumatoid arthritis (RA) FLSs were stimulated with 40 nM phosphatidylserine equivalents of MPs isolated either from THP-1 cells (MP THP-1) or from LPS-activated THP-1 cells (MP THP-1 LPS). Culture supernatants were harvested 24 hours after stimulation for IL-6 **(a) **and IL-8 **(b) **determination and 72 hours after stimulation for BAFF secretion **(c) **by enzyme-linked immunosorbent assay. LPS (IL-6 and IL-8) and interferon-gamma (IFN-γ) (BAFF) were used as positive controls. Data are expressed as the mean of triplicate samples ± standard deviation and are representative of three independent experiments. C, control medium; TMP, control Hanks' balanced saline solution.

As monocytes/macrophages are considered major instigators of joint inflammation, we also analyzed whether MPs isolated from LPS-activated THP-1 cells first stimulate IL-6 and IL-8 release by RA FLSs. As shown in Figure 4a,b, such MPs induced IL-6 and IL-8 release to a similar extent (3,000 ± 350 pg/mL) in RA FLS supernatants. An induction of BAFF release was observed after incubation with MPs (40 nM PhtdSer Eq) derived from LPS-activated THP-1 cells (Figure 4c). BAFF release could not be observed in negative controls.

### Microparticles promoted the synthesis of thymic stroma lymphopoietin and secretory leukocyte protease inhibitor by activated rheumatoid arthritis fibroblast-like synoviocytes

**Figure 5:**
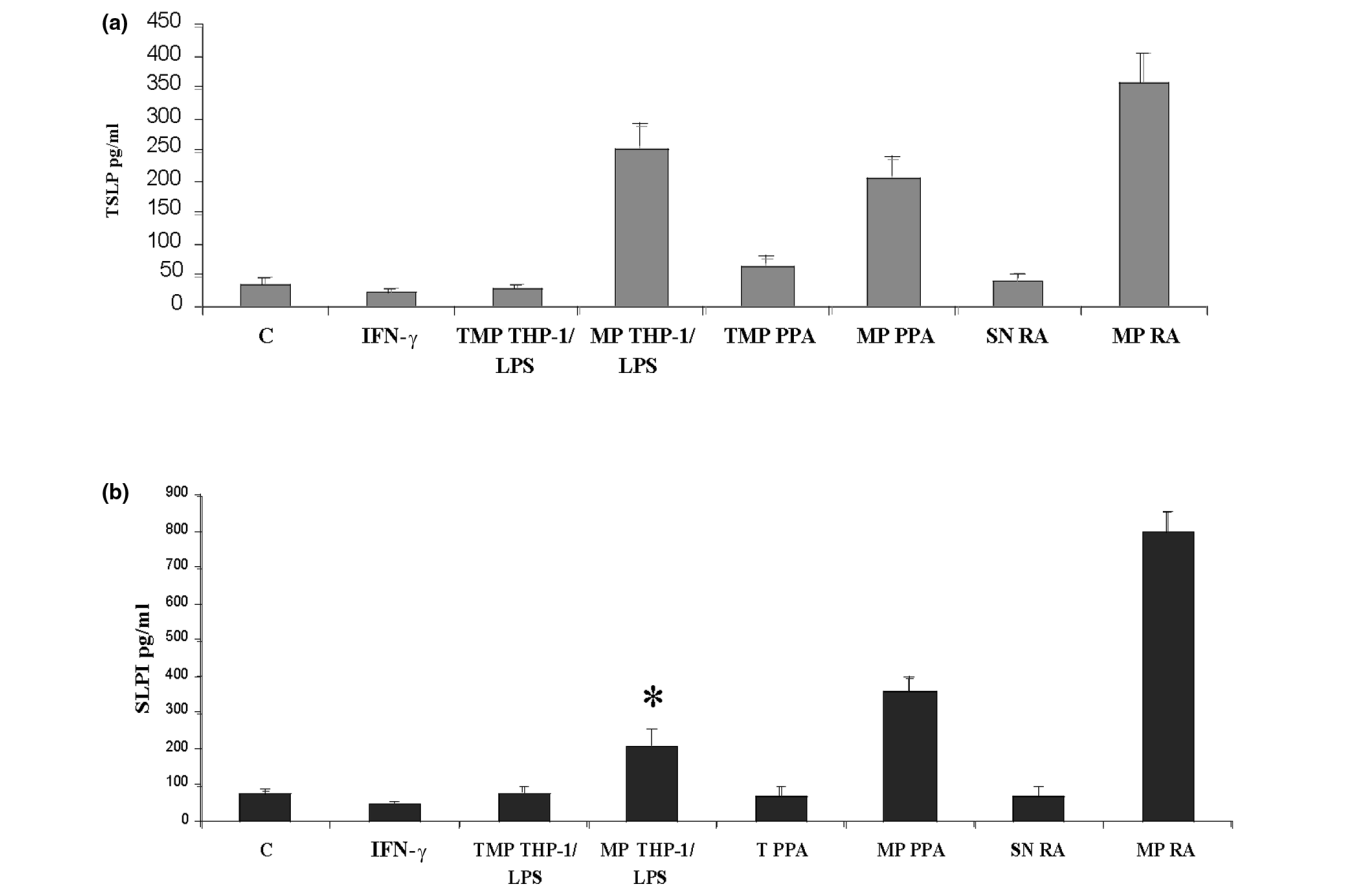
Induction of thymic stroma lymphopoietin (TSLP) and secretory leukocyte protease inhibitor (SLPI) synthesis by activated rheumatoid arthritis (RA) fibroblast-like synoviocytes (FLSs). TSLP **(a) **and SLPI **(b) **release was determined by enzyme-linked immunosorbent assay in supernatants of RA FLSs stimulated 48 hours (TSLP) and 72 hours (SLPI) with microparticles (MPs) (40 nM phosphatidylserine equivalents) isolated from RA synovial fluids, lipopolysaccharide (LPS)-treated THP-1 cells, and PHA, PMA and Adt-D (PPA)-treated CCRF-CEM cells. Data are expressed as the mean of triplicate samples ± standard deviation and are representative of three independent experiments. *P < 0.05. C, control medium; IFN-γ, interferon-gamma; SN, control supernatants; TMP, control Hanks' balanced saline solution.

Having found that FLSs released BAFF in response to MP, we sought to determine whether MP-activated FLSs could amplify B-cell activation by stimulating dendritic cells to produce BAFF via TSLP. RA FLSs were stimulated with MPs derived from synovial fluids, CEM lymphocytes, and THP-1 cells at the concentration of 40 nM PhtdSer Eq for 48 hours. As shown in Figure 5, RA FLSs released soluble TSLP protein after exposure to MPs. There were no significant differences between FLSs activated by MPs derived from synovial fluids, CEM lymphocytes, and THP-1 cells. SLPI is an antiprotease that is known to prevent BAFF-dependent class switching by inactivating nuclear factor-kappa-B (NF-κB). We detected SLPI in the supernantant of MP-activated RA FLSs 72 hours after activation. Thus, FLSs produce this homeostatic regulator of class switching after sensing MPs. Taken together, these results demonstrate that MPs derived either from synovial fluids from RA and OA patients or from leukocytes are able to induce BAFF, TSLP, and SLPI release by activated RA FLSs and could participate in cell-cell interactions leading to the proinflammatory response of FLSs as well as in their implication in the B-cell autoimmune response.

## Discussion

BAFF is a cytokine that plays a pivotal role in B-cell survival, differentiation, and activation. We and others recently demonstrated that resident cells of the synovial membrane synthesize and release BAFF in response to stimulation of innate immune receptors such as integrin α5β_1_ and IFN-γ receptors [20,21]. Beside this established key BAFF, other molecules might influence directly or indirectly B cells such as TSLP and SLPI. Direct evidence of the capacity of these factors to promote B-cell activation was demonstrated following TLR activation of oral epithelial cells [22].

As an increasing body of evidence suggested that MPs have potent proinflammatory activities and are potentially important mediators of inflammatory and autoimmune diseases [29-33], we have investigated in this study the role of MPs on BAFF, TSLP, and SLPI secretion by FLSs isolated from RA patients. MPs are produced during cell death but they may also arise during cell activation. They can be produced by virtually all cell types, but in contrast to MPs isolated from blood, MPs isolated from synovial fluids from RA patients are derived mainly from inflammatory and immune cells [23].

We first evaluated MP levels in synovial fluids obtained from patients with RA (7), OA (5), MC (3), and AR (5). All synovial fluids contained MPs although their levels seemed to be higher in RA and MC synovial fluids. These results are in accordance with observations indicating that the number of MPs is increased during inflammatory states *in vivo *[23].

As Distler and colleagues [16] demonstrated that MPs serve as important triggering elements to promote cytokine, chemokine, and MMP release from RA synovial fibroblasts, we then explored the role of RA and OA synovial fluid-derived MPs in inducing BAFF synthesis by activated FLSs. In the present study, we demonstrated a new mechanism by which FLSs could contribute to the adaptative autoimmune response. We showed that MPs, which are produced in synovial fluids during RA and OA, are potent stimuli of BAFF synthesis in a similar degree to IFN-γ, which is used as positive control. To our knowledge, this is the first time that BAFF induction by MPs has been demonstrated. This effect was observed with both RA and OA MPs, suggesting that MPs isolated from joints of patients with degenerative joint diseases such as OA have the same effect as MPs present in the joints of patients with RA and therefore that this action is not disease-dependent. The main difference regarding the assessed effect concerns the levels of MPs, which are much lower in synovial fluids of OA than of RA. However, it can be speculated that other types of cytokines and other functional effects of MPs which were not assessed in the present study might be different. It must be noted that, in contrast to other studies, this work was performed with purified MPs free of exosomes, which are preformed vesicles of endosomal origin which are stored intracellularly in multivesicular bodies and released by exocytosis. Exosomes, which are investigated mainly in the regulation of immune responses, do not expose phosphatidyserine, they share a common set of membrane molecules like tetraspanins, and they harbor unique subsets of proteins linked to cell type-associated functions [34,35]. Results suggest that MPs could contribute to the interplay between FLSs and B cells through BAFF synthesis. This induction of BAFF might also contribute to the increased proliferation of FLSs in RA, which might also be related to the autocrine effect of BAFF on FLSs. Indeed, FLSs not only secrete BAFF, they also bear BAFF receptors [36]. Berckmans and colleagues [15,37] found that, in patients with RA, most of the MPs present in the synovial fluid are produced by monocytes/macrophages, T cells, and granulocytes. MPs deriving from B cells, platelets, and erythrocytes are present only in low numbers.

To gain further information about the parental cells possibly involved in MP-mediated BAFF synthesis, we explored the effect of MPs isolated from CEM lymphocyte and THP-1 cells activated under various conditions. As previously shown by studies performed with LPS-treated U937 cells [38], THP-1 cell-derived MPs exhibit strong proinflammatory activities. Moreover, they were able to induce BAFF release by activated FLSs. In view of the abundance of these cells in the synovial cavity in RA, our results suggest that macrophages could serve as important triggering elements in promoting the inflammatory response and cooperation with B cells through the release of MPs. In contrast, we observed that MPs derived from activated CEM lymphocytes, which are inducers of IL-6 and IL-8 release, did not promote BAFF synthesis. BAFF release was not observed even with a 10-fold increase in MP concentration. T cells also occur abundantly in synovium and synovial fluids; nevertheless, our data demonstrate that MPs eventually produced by activated lymphocytes T *in vivo *cannot be considered key contributors in the induction of BAFF. We cannot rule out that MPs released by other cells present in the synovial cavity such as FLSs could interfere in this process. In fact, we have performed some preliminary experiments with MPs isolated from LPS-activated FLSs and have observed that these MPs act in an autocrine pathway and induce IL-6 release by activated FLSs (data not shown).

We reported also that MPs isolated from synovial fluids, CEM lymphocytes, or THP-1 cells were strong inducers of TSLP. TSLP is an IL-7-like cytokine that stimulates dendritic cells to produce more BAFF and constitutes a Th2-independent pathway for antibody production. It was demonstrated that epithelial cells lining tonsillar crypts released AID (activation-induced cytidine deaminase)-inducing factors, including BAFF, IL-10, and TSLP, after sensing viral RNA through TLR3 [22]. The resultant class switching caused the production of broadly IgG and IgA antibodies, including antibodies to self antigens. RA FLSs also release TSLP in response to LPS and poly I:C, and this effect is downregulated by IFN-γ and dexamethasone [39]. Our findings indicate that, like LPS and poly I:C, MPs could indirectly participate in B-cell activation by activating dendritic cells through TSLP release by RA FLSs and may be involved in the physiopathology of inflammatory arthritis.

However, we also showed that RA FLSs activated with MPs isolated from synovial fluids, CEM lymphocytes, or THP-1 cells release SLPI. SLPI was originally identified as a protein synthesized by macrophages which antagonized LPS activation of NF-κB. In B lymphocytes, SLPI inhibits class switching by interfering with NF-κB-dependent pathways and with the upregulation of AID induced by BAFF and viral RNA. As SLPI is released at a later time point, it may restrain the intrasynovial production of potentially pathogenic IgG antibodies. This needs to be demonstrated.

## Conclusions

Our studies indicated that MPs are potent inducers of proinflammatory factors as well as B-cell survival and promote the release of activation factors such as BAFF or TSLP by RA FLSs. This cellular response is regulated by SLPI release. Thus, MPs might play a fundamental role and behave as sensors in the control of humoral B-cell responses in RA synovium.

## Abbreviations

ActD: actinomycin D; AID: activation-induced cytidine deaminase; AR: reactive arthritis; BAFF: B cell-activating factor; ELISA: enzyme-linked immunosorbent assay; FCS: fetal calf serum; FLS: fibroblast-like synoviocyte; HBSS: Hanks' balanced saline solution; IFN-γ: interferon-gamma; IL: interleukin; LPS: lipopolysaccharide; MC: microcristalline arthritis; MCP: monocyte chemoattractant protein; MMP: matrix metalloproteinase; MP: microparticle; NF-κB: nuclear factor-kappa-B; OA: osteoarthritis; PHA: phytohemagglutinin; PhtdSer Eq: phosphatidylserine equivalents; PMA: phorbolmyristate acetate; PPA: combined PHA, PMA and actinomycin-D; RA: rheumatoid arthritis; SLPI: secretory leukocyte protease inhibitor; TLR: Toll-like receptor; TSLP: thymic stroma lymphopoietin.

## Competing interests

The authors declare that they have no competing interests.

## Authors' contributions

GA participated in designing and performing all experiments and in drafting the manuscript. LM participated in designing and performing all experiments and in drafting the manuscript and collected patient samples. FT-O supervised the characterization of MPs and the production of cell-derived MPs of various origins and edited the manuscript. FZ performed cell culture and MP quantitative determinations in patients and cell supernatants. IL performed cell culture and MP quantitative determinations in patients and cell supernatants. JS conceived the study. J-MF assisted in designing the study. J-EG edited the manuscript. DW conceived the study and drafted and edited the manuscript. All authors read and approved the final manuscript.
